# Prevalence, clinical characteristics, and disease burden of chronic cough in Italy: a cross-sectional study

**DOI:** 10.1186/s12890-024-03095-6

**Published:** 2024-06-20

**Authors:** Raffaele Antonelli Incalzi, Antonio De Vincentis, Vicky W. Li, Ashley Martin, Danilo Di Laura, Eileen Fonseca, Helen Ding

**Affiliations:** 1 Università Campus Biomedico di Roma and Fondazione Policlinico Universitario Campus Bio-Medico, via Alvaro del Portillo, 200, 00128, Rome, Italy; 2 Oracle Life Sciences, Seattle, WA USA; 3 MSD Italia S.r.l., Rome, Italy; 4 Merck & Co., Inc., Rahway, NJ USA

**Keywords:** Chronic cough, Cough, Chronic disease, Italy, Health surveys

## Abstract

**Background:**

Chronic cough has been associated with reduced health-related quality of life, negative impacts on sleep, work, and other daily activities, and increased use of health care resources. Little is known about the prevalence of chronic cough in Italy. In the present study we sought to estimate the prevalence of chronic cough in Italy, describe sociodemographic and clinical characteristics associated with chronic cough, and characterize the impact of chronic cough on overall health and wellness, work and other daily activities, and health care resource use.

**Methods:**

We conducted a cross-sectional study to collect sociodemographic and health-related data from Italian residents who participated in the 2020 National Health and Wellness Survey (*N* = 10,026). To assess the characteristics and burden of chronic cough, adults who indicated that they had experienced chronic cough during the prior 12 months were compared with propensity score-matched controls without chronic cough.

**Results:**

The estimated weighted lifetime and 12-month prevalence of chronic cough were estimated as 9.2% and 6.3%, respectively. Compared with matched controls, respondents with chronic cough had significantly lower measures of overall physical and mental health (*P* < .001 for both comparisons), and significantly higher rates of anxiety, depression, and sleep disorders (*P* < .001 for all comparisons). Chronic cough was significantly associated with higher rates of impairment of work and other activities (*P* < .001 for all comparisons) in the past 7 days, any-cause emergency department visits and hospitalizations in the prior 6 months (*P* < .001 for both comparisons), and more visits to general and specialist health care providers (*P* < .001 for both comparisons) in the prior 6 months.

**Conclusions:**

In Italy, chronic cough affects an estimated 3.3 million adults annually and represents a significant burden to individuals and the health care system.

**Take home message:**

Little is known about the prevalence of chronic cough in Italy. We found that, in Italy chronic cough represents a significant burden to individuals and the health care system, affecting an estimated 3.3 million adults annually.

**Supplementary Information:**

The online version contains supplementary material available at 10.1186/s12890-024-03095-6.

## Background

Globally, approximately 10% of adults have chronic cough [1], a protracted and debilitating condition, in which over 40% of affected adults report living with this condition for ≥ 5 years [2, 3]. A 2015 meta-analysis of global studies found that the burden of chronic cough is highest in North America and Europe, the latter having an estimated prevalence of 12.7% [1]. Factors associated with chronic cough in clinic- and population-based studies from Europe include smoking, female sex, and, to a lesser extent, older age [4–10]. However, the findings of clinic-based studies may not be generalizable due to differences in cough severity and health utilization patterns. Furthermore, population-based studies from Europe do not use a consistent definition of chronic cough, and prevalence estimates for individual European countries are frequently limited in number [1, 11]. Current estimates of chronic cough prevalence in Italy are based on studies with limited sample sizes primarily conducted during the 1980s and 1990s [4, 5, 12].

In the current study, we sought to address this knowledge gap by using a nationally representative survey to estimate the 12-month and lifetime prevalence of chronic cough among Italian adults. We further sought to describe the sociodemographic factors, comorbidities, and health care utilization patterns associated with chronic cough in Italy.

## Methods

### Study design

An analysis of chronic cough in Italy was carried out using data from the National Health and Wellness Survey (NHWS). The NHWS is an international, self-administered, internet-based health survey conducted annually in Italy, other countries in Europe and Asia, and the United States. The data reported here were derived from the 2020 fielding of the NHWS, which was administered to an age- and sex-representative sample of adults in Italy from December 2019 through March 2020. The prevalence of chronic cough was estimated in a cross-sectional analysis; factors associated with chronic cough were identified in a matched case-control analysis. All study respondents underwent informed consent, and respondent information was fully anonymized. The study (protocol, 19-KANT-205) received an exempt status upon evaluation by the Pearl Institutional Review Board.

### Study sample

Respondents to the NHWS were recruited via an existing general-purpose, voluntary, web-based survey. Inclusion criteria were age ≥ 18 years at the time of the survey, literacy in Italian, and the ability to provide informed consent; there were no specific exclusion criteria. Respondents were stratified based on their history of chronic cough: those who indicated that they had ever experienced chronic cough (defined as daily cough for ≥ 8 weeks, in alignment with clinical guidelines [13–15]) were included in calculations of lifetime prevalence, whereas those who indicated experiencing chronic cough in the past 12 months were included in calculations of 12-month prevalence. Respondents who did not experience chronic cough in the past 12 months were retained as a control group for propensity score-matched comparisons.

### Study measures

Study measures have been detailed elsewhere [16]. Health-related quality of life was assessed using the Medical Outcomes Study 12-item Short Form Survey v2 (SF-12v2; Quality Metric, Lincoln, RI [17]) and the EuroQol 5-Dimension Health Questionnaire (EQ-5D-5 L^™^; EuroQol Research Foundation) [18]. Three summary measures derived from the SF-12v2 were used in the NHWS: the physical and mental health components of the SF-12v2 (calculated using a mean score of 50 and a standard deviation (SD) of 10) and the SF-6D, a preference-based health utility index. For all three measures, higher scores represent better health. The composite index score was reported for the EQ-5D descriptive system, with 0 representing death and 1 the best possible health state. The EQ-5D also includes a vertical visual analogue scale (VAS), asking respondents to indicate on a number line their self-rated health for that day with 0 representing the “worst imaginable health state” and 100 representing the “best imaginable health state”. The General Anxiety Disorder 7-item scale (GAD-7) was used to assess anxiety [19], and the Patient Health Questionnaire 9-item scale (PHQ-9) was used to assess depression [20]. Both the GAD-7 and the PHQ-9 measure symptom frequency during the prior 2 weeks using a Likert scale ranging from 0 (not at all) to 3 (nearly every day). Total scores for the GAD-7 range from 0 to 21, with values 15 and above indicating severe anxiety symptoms. Total scores for the PHQ-9 range from 0 to 27, with values 20 and above indicating severe depression.

Health-related impairment of work and activity during the past 7 days was measured using the Work Productivity and Activity Impairment (WPAI) [21]. The 6-item WPAI measures four impairment outcomes: the percentage of work time missed due to health (absenteeism), the percentage of working hours impaired due to health (presenteeism), total work productivity loss due to health (productivity impairment), and the percentage of non-work activity impairment due to health (activity impairment). All respondents provided data for activity impairment; only respondents who reported being employed provided data on work-related impairments.

Difficulty sleeping was evaluated with yes or no questions regarding whether the respondent regularly experienced the following: difficulty falling asleep, waking during the night and not being able to get back to sleep, waking up several times during the night, waking up earlier than desired, sleep apnea, poor quality of sleep, daytime sleepiness, or difficulty staying awake.

All-cause health care resource use was measured as the number of hospitalizations and visits to health care practitioners (general or family clinicians, specialists, or emergency department [ED] clinicians) made in the past 6 months.

### Data analysis

The prevalence of chronic cough was calculated using unweighted estimates, as well as weighted estimates adjusted for the age and sex distribution of the Italian population. The unweighted estimates of lifetime and 12-month prevalence of chronic cough were calculated using the total number of NHWS respondents as the denominator. The weighted prevalence estimates were generated by applying Horvitz-Thompson sampling weights calculated using the age and sex distributions reported for Italy in the 2019–2020 International Data Base of the United States Census Bureau [22].

Comparisons were conducted between adults with chronic cough and matched controls without chronic cough using propensity score matching. Adults who experienced chronic cough within the past 12 months were compared with a propensity score-matched sample of adults without chronic cough from the general population (i.e., those who did not experience chronic cough within the past 12 months) using a 1:3 matching ratio. Logistic regression modeling was used to estimate propensity scores. Explanatory variables included in the logistic model were age, sex, marital status, household income, an interaction term for marital status x household income, and a modified Charlson Comorbidity Index (CCI) that excluded chronic obstructive pulmonary disease (COPD), as this condition is commonly associated with chronic cough. The variables were selected for matching based on clinical importance/interest or those deemed to be potential confounders to account for differences in health care and general health status between adults of different sexes, age, and socioeconomic status and comorbid conditions not associated with chronic cough. Data were reported as means and SD for continuous variables, and frequency counts and percentages for categorical variables. Statistical comparisons were conducted using chi-square tests and t-tests, as appropriate. Two-tailed *P* values of < 0.05 were considered statistically significant.

## Results

### Prevalence of chronic cough in Italy

Among all the 10,026 NHWS survey respondents, 905 (9.0%) reported experiencing chronic cough during their lifetime and 619 (6.2%) respondents reported experiencing chronic cough during the previous 12 months (Table 1). The weighted analysis resulted in similar findings, with the lifetime and 12-month prevalence rates of chronic cough in Italy’s adult population estimated to be 9.2% and 6.3% or 4.8 million and 3.3 million adults, respectively.

**Table 1:** Lifetime and 12-month prevalence of chronic cough among italian adults

	Lifetime prevalence						12-month prevalence					
Population	Unweighted			Weighted^a^			Unweighted			Weighted^a^		
	N	n (%)	*P* value	N	n (%)	*P* value	N	n (%)	*P* value	N	n (%)	*P* value
Total population	10,026	905 (9.0)		52,136,036	4,778,171 (9.2)		10,026	619 (6.2)		52,136,036	3,280,157 (6.3)	
Sex			0.009			< 0.001			< 0.001			< 0.001
Female	5,229	542 (10.4)		27,300,869	2,816,316 (10.3)		5,229	385 (7.4)		27,300,869	2,053,903 (7.5)	
Male	4,797	363 (7.6)		24,835,167	1,961,855 (7.9)		4,797	234 (4.9)		24,835,167	1,226,254 (4.9)	
Age group (years)			< 0.001			0.452			0.308			0.484
18–29	1,463	142 (9.7)		7,564,987	716,810 (9.5)		1,463	85 (5.8)		7,564,987	411,313 (5.4)	
30–39	1,488	153 (10.3)		7,575,594	759,897 (10.0)		1,488	90 (6.0)		7,575,594	443,196 (5.9)	
40–49	1,909	191 (10.0)		9,777,645	957,186 (9.8)		1,909	131 (6.9)		9,777,645	651,963 (6.7)	
50–64	2,569	224 (8.7)		13,585,138	1,192,947 (8.8)		2,569	162 (6.3)		13,585,138	874,276 (6.4)	
65–74	2,233	161 (7.2)		11,872,569	981,270 (8.3)		2,233	122 (5.5)		11,872,569	755,925 (6.4)	
≥ 75	364	34 (9.3)		1,760,103	170,061 (9.7)		364	29 (8.0)		1,760,103	143,484 (8.2)	
Smoking status			< 0.001			< 0.001			< 0.001			< 0.001
Current / former	5,777	633 (11.0)		29,736,076	3,331,547 (11.2)		5,777	448 (7.8)		29,736,076	2,359,280 (7.9)	
Never	4,249	272 (6.4)		22,399,960	1,446,624 (6.5)		4,249	171 (4.0)		22,399,960	920,877 (4.1)	
Region			< 0.001			< 0.001			< 0.001			- ^b^
Center	2,175	192 (8.8)		11,354,446	1,014,812 (8.9)		2,175	127 (5.8)		11,354,446	678,316 (6.0)	
North	4,837	364 (7.5)		25,089,168	1,908,159 (7.6)		4,837	249 (5.1)		25,089,168	1,315,447 (5.2)	
South	3,009	348 (11.6)		15,671,826	1,851,199 (11.8)		3,009	243 (8.1)		15,671,826	1,286,394 (8.2)	
Other	5	1 (20.0)		20,596	4,001 (19.4)		5	0 (0.0)		20,596	0 (0.0)	

^a^  The prevalence of chronic cough was calculated using unweighted estimates, as well as weighted estimates adjusted for the age and sex distribution of the Italian population. The unweighted estimates of lifetime and 12-month prevalence of chronic cough were calculated using the total number of NHWS respondents as the denominator. The weighted prevalence estimates were generated by applying Horvitz-Thompson sampling weights calculated using the age and sex distributions reported for Italy in the 2019–2020 International Data Base of the United States Census Bureau [22]

^b^ The weighted *P* values cannot be estimated due to the empty cell in the ‘Other’ category

In the weighted analysis, the lifetime prevalence of chronic cough was higher among females compared with males (10.3% vs. 7.9%; *P* < .001; Table 1) and among respondents with a history of smoking compared with those who had never smoked (11.2% vs. 6.5%; *P* < .001). Regional differences in the lifetime prevalence of chronic cough were also observed (*P* < .001), with a higher prevalence in southern Italy (11.8%) than in central (8.9%) and northern (7.6%) Italy. Trends in the 12-month prevalence of chronic cough were similar to those of lifetime prevalence. The 12-month prevalence of chronic cough was higher in females compared with males (7.5% vs. 4.9%; *P* < .001) and in former or current smokers compared with never smokers (7.9% vs. 4.1%; *P* < .001). Unweighted lifetime and 12-month prevalence estimates were similar to weighted estimates, varying by less than 0.4% in 90% of strata.

### Sociodemographic characteristics of respondents with chronic cough

A total of 614 respondents with chronic cough were matched (five of the 619 respondents with chronic cough were not matched; four respondents had only two matches and another three respondents had only one match). Thus, the total number of matched controls without chronic cough is 1,832.

In the propensity score-matched analysis, respondents with chronic cough differed from matched controls by level of education (*P* = .026) and region of residence (*P* < .001; Supplementary Table 1). As expected, due to the matching, there is no significant difference between respondents with chronic cough compared with propensity score - matched controls in distributions of age, sex, household income and marital status; employment status and type of insurance also did not significantly differ between respondents with chronic cough and propensity score-matched controls.

### Health and health-related quality of life impacts of chronic cough

Respondents with chronic cough during the past 12 months reported poorer health-related quality of life compared with propensity score-matched controls without chronic cough, as shown by lower mean values of the physical (47.22 vs. 50.19) and mental (40.68 vs. 44.83) component summary scores of the SF-12v2 and the SF-6D utility score (0.63 vs. 0.68; *P* < .001 for all comparisons; Table 2). Further, EQ-5D scores were lower, indicating a worse health state, for those with chronic cough compared to those without (0.83 vs. 0.88; *P* < .001) and VAS (65.70 vs. 73.72; *P* < .001). Compared with propensity score-matched controls without chronic cough, respondents with chronic cough more frequently reported digestive (38.8% vs. 23.7%), respiratory (64.2% vs. 24.8%), and sleep (31.3% vs. 14.4%) conditions during the past 12 months (*P* < .001 for all comparisons).

**Table 2:** Health characteristics of respondents with and without chronic cough in the previous 12 months

Health characteristic	Respondents			*P* value
	Total matched population (*N* = 2,446)	Chronic cough (*N* = 614)	No chronic cough (*N* = 1,832)	
CCI (modified—without COPD)				0.174
0 comorbidities	1,809 (74.0)	437 (71.2)	1,372 (74.9)	
1 comorbidity	373 (15.2)	106 (17.3)	267 (14.6)	
≥ 2 comorbidities	264 (10.8)	71 (11.6)	193 (10.5)	
SF-12v2 physical component summary score ^a^				**< 0.001**
Mean (SD)	49.45 (8.55)	47.22 (8.38)	50.19 (8.47)	
SF-12v2 mental component summary score ^a^				**< 0.001**
Mean (SD)	43.79 (9.52)	40.68 (9.28)	44.83 (9.38)	
SF-6D utility score ^b^				**< 0.001**
Mean (SD)	0.67 (0.11)	0.63 (0.10)	0.68 (0.12)	
EQ-5D-3 L, mean (SD)				**< 0.001**
EQ-5D index ^c^	0.86 (0.11)	0.83 (0.11)	0.88 (0.11)	
EQ-5D VAS ^d^	71.71 (22.06)	65.70 (21.60)	73.72 (21.86)	
GAD-7 anxiety score				**< 0.001**
0–4: no anxiety	1,062 (43.4)	169 (27.5)	893 (48.7)	
5–9: mild anxiety	955 (39.0)	277 (45.1)	678 (37.0)	
10–14: moderate anxiety	287 (11.7)	110 (17.9)	177 (9.7)	
15–21: severe anxiety	142 (5.8)	58 (9.4)	84 (4.6)	
PHQ-9 depression score				**< 0.001**
0–4: none–minimal	1,059 (43.3)	170 (27.7)	889 (48.5)	
5–9: mild depression	798 (32.6)	218 (35.5)	580 (31.7)	
10–14: moderate depression	345 (14.1)	120 (19.5)	225 (12.3)	
15–19: moderately severe depression	169 (6.9)	69 (11.2)	100 (5.5)	
20–27: severe depression	75 (3.1)	37 (6.0)	38 (2.1)	
Medical conditions diagnosed in prior 12 months				
Cancer	87 (3.6)	17 (2.8)	70 (3.8)	0.476
Chronic pain condition ^e^	128 (5.2)	40 (6.5)	88 (4.8)	0.257
Digestive condition ^f^	673 (27.5)	238 (38.8)	435 (23.7)	**< 0.001**
Current medication ^g^	396 (58.8)	150 (63.0)	245 (56.6)	0.103
Respiratory condition ^h^	848 (34.7)	394 (64.2)	454 (24.8)	**< 0.001**
Current medication ^g^	433 (51.1)	210 (53.3)	223 (49.1)	0.225
Sleep condition ^i^	455 (18.6)	192 (31.3)	263 (14.4)	**< 0.001**
Current medication ^g^	198 (43.5)	80 (41.7)	118 (44.9)	0.496

CCI, Charlson Comorbidity Index; COPD, chronic obstructive pulmonary disease; EQ-5D-5L, EuroQol 5-Dimension Health Questionnaire; SD, standard deviation; SF-12v2, Medical Outcomes Study 12-item Short Form Survey Version 2; VAS, visual analogue scale. Chronic cough was defined as daily cough for ≥ 8 weeks. Values are presented as n (%) unless otherwise stated. Statistically significant *P* values (< 0.05) are shown in **bold**

^a^ The overall mean (SD) score for this survey was set at 50 (10). Higher scores indicate better health

^b^ The SF-6D health utility score (on a scale of 0 to 1, with 1 representing perfect health) is derived from participant responses to the SF-12v2

^c^ The composite index score was reported for the EQ-5D descriptive system, with 0 representing death and 1 the best possible health state

^d^ The EQ-5D vertical visual analogue scale (VAS), asked respondents to indicate on a number line their self-rated health for that day with 0 representing the “worst imaginable health state” and 100 representing the “best imaginable health state”

^e^ All positive responses in this category involved rheumatoid arthritis

^f^ This category represents respondents who reported experiencing one or more digestive conditions from a list provided (gastroesophageal reflux disease, heartburn, irritable bowel syndrome, ulcerative colitis, ulcers)

^g^ ‘Current medication’ indicates the number (%) of respondents who were taking prescription medication for the indicated medical condition at the time of the survey

^h^ This category represents respondents who reported experiencing one or more respiratory conditions from a list provided (allergies, asthma, hay fever, chronic bronchitis, chronic cough, COPD, emphysema)

^i^ This category represents respondents who reported experiencing one or more sleep conditions from a list provided (insomnia, narcolepsy, sleep apnea, other sleep problems)

### Health care resource use burden of chronic cough

In total, 94.1% of respondents with chronic cough reported the use of health care provider services for any reason during the prior 6 months compared with 87.0% of propensity score-matched controls (*P* < .001; Table 3). On average, respondents with chronic cough had two more visits with any health care provider during the prior 6 months than did the matched controls (7.84 vs. 5.46; *P* < .001). Compared with propensity score-matched controls, respondents with chronic cough more frequently reported hospitalization (12.5% vs. 7.4%), the use of ED care (23.0% vs. 12.9%), the use of specialist care provided by general or family practitioners (75.9% vs. 69.8%; *P* < .004), and visits to allergists (11.4% vs. 4.9%), cardiologists (20.5% vs. 13.6%), gastroenterologists (9.6% vs. 5.6%), geriatricians (1.6% vs. 0.2%), otolaryngologists (7.2% vs. 3.8%), and pulmonologists (9.3% vs. 2.0%; *P* < .001 for all comparisons except general or family practitioners *P* = .004). Significant differences between groups in the use of services provided by internists, neurologists, psychiatrists, or psychologists/therapists were not observed.

**Table 3:** Health care resource use in prior 6 months by respondents with and without chronic cough in the previous 12 months

Health care resource visit type	Respondents using each resource in prior 6 months *n* (%)				Number of visits per respondent in prior 6 months Mean (SD)			
	Total matched population (*N* = 2,446)	Chronic cough (*N* = 614)	No chronic cough (*N* = 1,832)	*P* value	Total matched population (*N* = 2,446)	Chronic cough (*N* = 614)	No chronic cough (*N* = 1,832)	*P* value
Hospitalization	213 (8.7)	77 (12.5)	136 (7.4)	**< 0.001**	0.16 (0.80)	0.28 (1.24)	0.12 (0.58)	**0.002**
Emergency department	377 (15.4)	141 (23.0)	236 (12.9)	**< 0.001**	0.29 (0.92)	0.44 (1.05)	0.24 (0.86)	**< 0.001**
Visited any health care provider	2,172 (88.8)	578 (94.1)	1,594 (87.0)	**< 0.001**	6.06 (7.39)	7.84 (9.30)	5.46 (6.53)	**< 0.001**
Visited any specialist	1,924 (78.7)	530 (86.3)	1,394 (76.1)	**< 0.001**	3.62 (5.23)	4.61 (5.91)	3.29 (4.93)	**< 0.001**
Visited any of the below specialties								
Allergist	159 (6.5)	70 (11.4)	89 (4.9)	**< 0.001**	0.10 (0.54)	0.16 (0.60)	0.08 (0.52)	**0.004**
Cardiologist	375 (15.3)	126 (20.5)	249 (13.6)	**< 0.001**	0.21 (0.65)	0.30 (0.80)	0.18 (0.59)	**0.001**
Gastroenterologist	161 (6.6)	59 (9.6)	102 (5.6)	**< 0.001**	0.09 (0.41)	0.13 (0.46)	0.08 (0.39)	**0.016**
General / family practitioner	1,744 (71.3)	466 (75.9)	1,278 (69.8)	**0.004**	2.58 (3.70)	3.10 (3.82)	2.40 (3.65)	**< 0.001**
Geriatrician	13 (0.5)	10 (1.6)	3 (0.2)	**< 0.001**	0.01 (0.11)	0.02 (0.13)	0.00 (0.11)	**0.029**
Internist	46 (1.9)	19 (3.1)	27 (1.5)	0.110	0.03 (0.25)	0.05 (0.32)	0.02 (0.22)	0.116
Neurologist	146 (6.0)	42 (6.8)	104 (5.7)	0.292	0.11 (0.78)	0.15 (1.29)	0.09 (0.51)	0.271
Otolaryngologist	114 (4.7)	44 (7.2)	70 (3.8)	**0.001**	0.07 (0.34)	0.09 (0.35)	0.06 (0.34)	**0.042**
Psychiatrist	71 (2.9)	24 (3.9)	47 (2.6)	0.086	0.09 (0.91)	0.15 (1.27)	0.08 (0.75)	0.175
Psychologist / Therapist	86 (3.5)	24 (3.9)	61 (3.3)	0.388	0.28 (2.11)	0.31 (2.61)	0.27 (1.91)	0.711
Pulmonologist	94 (3.8)	57 (9.3)	37 (2.0)	**< 0.001**	0.06 (0.40)	0.16 (0.67)	0.03 (0.23)	**< 0.001**

SD, standard deviation. Chronic cough was defined as daily cough for ≥ 8 weeks. Statistically significant *P* values (< 0.05) are shown in **bold**

### Impact of chronic cough on impairment of work and other activities

**Fig. 1:**
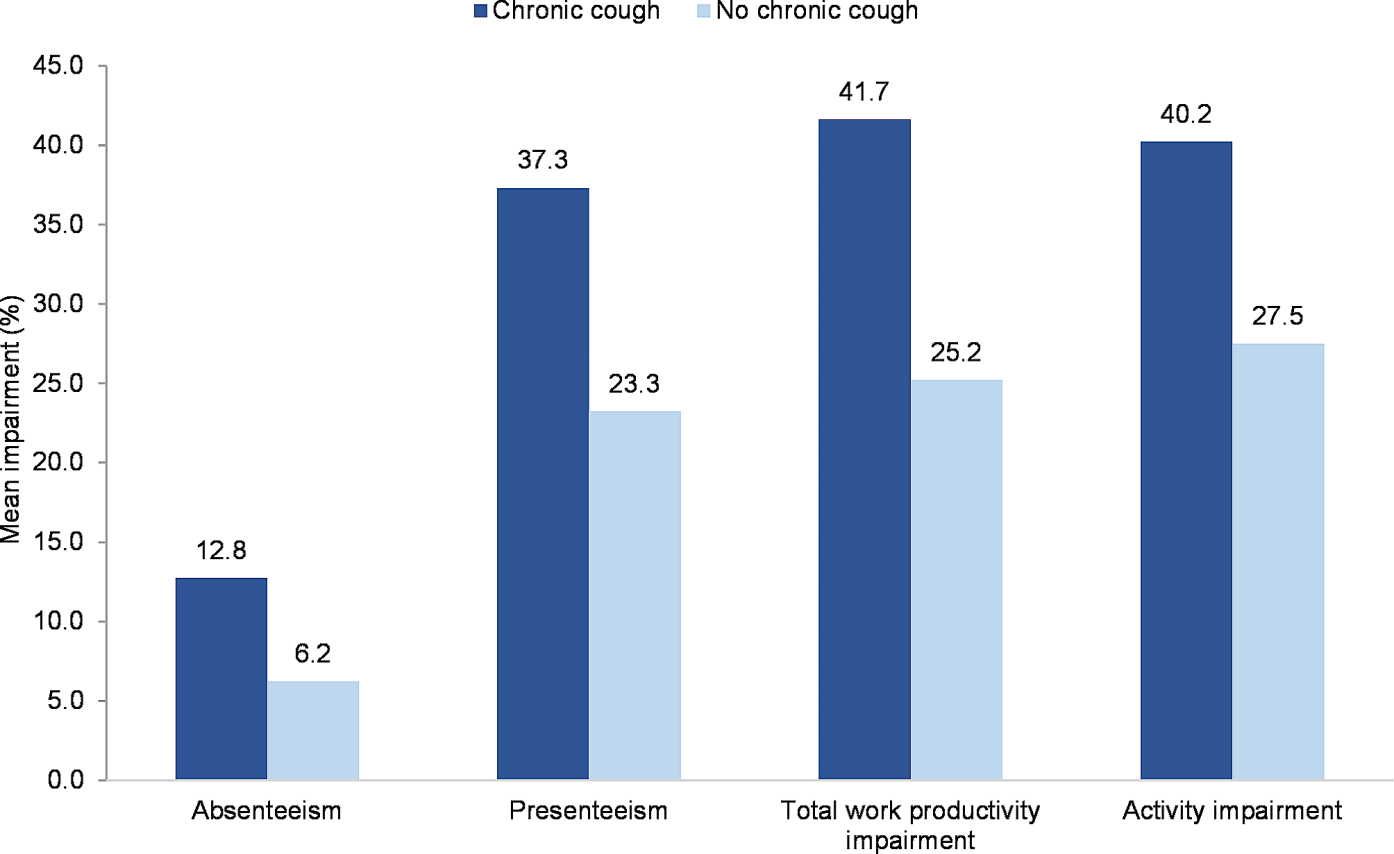
Impairment of work and other daily activities in respondents with and without chronic cough in the previous 12 months. Chronic cough was defined as daily cough for ≥ 8 weeks. The bars represent absenteeism (mean % missed work hours; employed respondents only), presenteeism (mean % impaired work hours; employed respondents only); total work productivity impairment (mean % combined impact of absenteeism and presenteeism; employed respondents only), and activity impairment (mean % impairment of non-paid activities; all respondents) during the prior 7 days. Differences between the ‘chronic cough’ and ‘no chronic cough’ groups were statistically significant (*P* < .001) for all four measures

Compared with propensity score-matched controls, respondents with chronic cough reported more work impairment, including absenteeism (mean % of missed working hours: 12.8% vs. 6.2%), presenteeism (mean % of impaired working hours: 37.3% vs. 23.4%), and impaired total work productivity (mean %: 41.7% vs. 25.2%; *P* < .001 for all comparisons; Fig. 1). Activity impairment was also more frequently reported by respondents with chronic cough than matched controls (mean %: 40.2% vs. 27.5%; *P* < .001).

## Discussion

There is a lack of data describing chronic cough and its impact in Italy and the present study contributes to this knowledge gap. In summary, we found that Italian adults have an appreciable burden of chronic cough, corresponding to an estimated weighted lifetime and 12-month prevalences of 9.2% and 6.3%, respectively. The prevalence of chronic cough was highest among females, current and former smokers, and residents of southern Italy. In an analysis adjusted for age, sex, marital status, household income, and a modified CCI, chronic cough was associated with residence in southern Italy and having less than a high school education. Notably, chronic cough was associated with significantly increased health care utilization, work and activity impairment, and poorer health outcomes, including more comorbidities, lower physical and mental health status, anxiety, and depression.

Information on the prevalence chronic cough in Italy is limited, however, our estimated prevalences (9.2% [lifetime]/6.3% [12-month]) are similar to those reported by authors using the same definition of chronic cough and comparable survey-based methods as the current study; including Denmark (3.8% 12-month) [8], France (7.5% [lifetime]/4.8% [12-month]) [23], Germany (6.5% [lifetime]/4.9% [12-month]) [24], Spain (8.2% [lifetime]/5.5% [12-month]) [25], the United Kingdom (6.2% [lifetime]/4.9% [12-month]) [26], and the United States (5.0% 12-month) [16]. In contrast, the prevalence of chronic cough in Italy was estimated to be in the range of 10–15% in a 2015 meta-analysis [1]. However, the three Italian studies included in the meta-analysis were conducted in the 1980s and 1990s and defined chronic cough as a cough lasting ≥ 3 months during the past two years [4, 5, 12]. In the current study we defined chronic cough as a daily cough ≥ 8 weeks duration, the definition which has been utilized in clinical guidelines [13–15]. Differences in the definition of chronic cough are unlikely to fully explain the lower prevalence of chronic cough reported in our study as studies using a threshold of 8 weeks to define chronic cough typically report higher prevalences than those using a threshold of 3 months [11]. It is possible that the burden of chronic cough among Italian adults, though still considerable, may have decreased from the 1980s and 1990s. This may be partially explained by changing demographics and cultural practices. For instance, smoking, an established risk factor for chronic cough [27], has decreased in Italy with male smoking prevalence decreasing from 41.6% in 1986 to 29.5% in 2009 [28] and female smoking prevalence decreasing from 19.2% in 1986 to 17.0% in 1993 [28].

We evaluated age- and sex-related differences in chronic cough, finding a significantly higher prevalence of chronic cough among females and a numerical trend of increasing prevalence of cough in the prior 12 months with older age. These findings align with those of European and North American studies of cough clinics, which report a preponderance of older women [29]. Our findings also align with those of most [5–8], but not all population-based studies from Europe [30], showing higher chronic cough prevalence with increasing age. In contrast, many [4, 6, 8, 30], but not all [7], population surveys from Europe report either no sex-related differences in chronic cough prevalence or a higher rate in males [5]. Mixed findings regarding sex-related trends in chronic cough may reflect sampling differences between clinic- and population-based studies, as well as complex epidemiology. Biological differences, including higher levels of estrogen and progesterone, may render women more susceptible to chronic cough, while cough risk factors, including smoking and occupational exposures, are higher among men than women in some settings [29].

In a propensity score-matched analysis controlling for age, sex, marital status, household income, and comorbidities, we found that chronic cough was associated with a lower education level. Several population-based studies support this finding, reporting an approximate 50% increase in the risk of chronic cough among individuals with a low compared with a high socioeconomic or educational level [4, 8, 9, 27]. Our propensity score-matched analysis also found a significantly higher prevalence of chronic cough among individuals residing in southern compared with central or northern Italy. Although evidence is scant regarding subnational differences in the burden of chronic cough, three studies from Italy have evaluated this issue. One study found sub-regional differences in the prevalence of chronic cough in Italy’s Po River valley [12]. Two other studies reported higher prevalences of asthma and respiratory symptoms in southern compared with central or northern Italy, which in quantitative analyses were primarily attributable to climatic factors [31, 32].

Our findings showed that Italian adults with chronic cough had an excess burden of comorbidities, including anxiety, depression, and sleep disorders. These findings accord with those of population-based surveys from Europe, including a multi-country survey in which over 90% of adults with chronic cough reported feeling depressed due to their cough [33]. The findings of these European surveys highlight ways in which chronic cough may undermine mental health by disrupting social interactions and engendering frustration over inadequate treatment for cough despite high health care utilization [33, 34].

We observed substantially greater health care utilization among Italian adults with chronic cough compared with adults without cough, including nearly twice the rate of ED visits and hospitalizations. Results of a 2020 fielding of the NHWS conducted in the United States similarly reported that adults with chronic cough visited the ED about twice as often as those without chronic cough [16]. Notably, the costs associated with respiratory disease are not limited to greater health care utilization, but also encompass impaired work productivity, particularly among individuals with severe symptoms. Indeed, we observed a striking burden of impaired work productivity associated with chronic cough in the previous 12 months, equating to over 40% of work hours. Recent studies from Asia, Europe, and the United States report a similar degree of work productivity impairment of 26-43% for adults with chronic cough [16, 23, 25, 26, 35, 36].

The current study has limitations that should be considered when interpreting the findings. The data reported here are derived from the 2020 fielding of the NHWS, which was administered during the early stages of the COVID-19 pandemic. As data on the SARS-CoV-2 respiratory virus continues to unfold, and its effects on the prevalence and characteristics of chronic cough are not yet known [37, 38]. Therefore, the prevalence of chronic cough reported here may differ from the prevalence of chronic cough in Italy before, during, or after phases of the COVID-19 pandemic. Recall bias and self-presentation bias may have affected study results, as data on study outcomes were collected using a self-administered questionnaire. However, survey questions and response options were phrased to minimize these biases. For example, where possible, questions referred to recent timeframes to enable more accurate recall. Potentially sensitive questions included uninformative response options, such as ‘Don’t Know’ and ‘Refuse to Answer’ that allowed respondents to opt out. Further, the confidential nature of this survey may also have limited self-presentation bias. We conducted an adjusted analysis by propensity matching on age, sex, household income, marital status, and comorbidities; however, residual confounding by other factors may have affected the results. Although we enrolled a nationally representative sample of Italian adults, study findings may not be generalizable to subgroups excluded from the study, including institutionalized individuals or those who have severe disabilities, or limited internet access. In addition, the structure of the study population was skewed toward younger adults, and individuals > 75 years were under-represented. The study’s cross-sectional design limits the use of its findings for making causal inferences regarding chronic cough.

## Conclusions

This study revealed a substantial burden of chronic cough among Italian adults. This burden included an estimated 12-month weighted prevalence of 6.3%, equivalent to over 3.3 million affected individuals, and an estimated weighted lifetime prevalence of 9.2%, equivalent to over 4.8 million affected individuals. This burden also manifested in greater rates of depression, anxiety, work impairment, and health care utilization among adults with chronic cough compared with their unaffected counterparts. Further research is warranted into the causes underlying the greater prevalence of chronic cough among women, individuals with lower education, and residents of southern Italy.

### Electronic supplementary material

Below is the link to the electronic supplementary material.

Supplementary Material 1

## Data Availability

The data that support the findings of this study are available from Oracle Life Sciences, Seattle, WA, USA but restrictions apply to the availability of these data, which were under license for the current study, and so are not publicly available.

## Abbreviations

CCI: Charlson Comorbidity Index
COPD: Chronic obstructive pulmonary disease
ED: Emergency department
EQ-5D-5L: EuroQol 5-Dimension Health Questionnaire
GAD-7: General Anxiety Disorder 7-item scale
NHWS: National Health and Wellness Survey
PHQ-9: Patient Health Questionnaire 9-item scale
SD: Standard deviation
SF-12v2: Medical Outcomes Study 12-item Short Form Survey v2
VAS: Visual analog scale
WPAI: Work Productivity and Activity Impairment
