# Supplementary material for: Prevalence, clinical characteristics, and disease burden of chronic cough in Italy: a cross-sectional study
Source: BMC Pulm Med. 2024 Jun 20;24:288. doi: 10.1186/s12890-024-03095-6 (PMC11191261; doi:10.1186/s12890-024-03095-6)
Supplement: Supplementary file 1 — Supplementary Material 1 [file 12890_2024_3095_MOESM1_ESM.docx]

Supplementary Table 1. Sociodemographic Characteristics of Respondents With and Without Chronic Cough in the Previous 12 Months

| Sociodemographic characteristic | Respondents | | | *P* value |
| --- | --- | --- | --- | --- |
|  | Total matched population  (N = 2,446) | Chronic cough  (N = 614) | No chronic cough  (N = 1,832) |  |
| Gender ^a^ |  |  |  | .524 |
| Male | 898 (36.7) | 232 (37.8) | 666 (36.4) |  |
| Female | 1,548 (63.3) | 382 (62.2) | 1,166 (63.6) |  |
| Mean age (years), mean (SD) | 49.9 (16.2) | 49.8 (15.9) | 50.0 (16.3) | .783 |
| Age group (years) ^a^ |  |  |  | .232 |
| 18–24 | 160 (6.5) | 31 (5.0) | 129 (7.0) |  |
| 25–39 | 532 (21.7) | 139 (22.6) | 393 (21.5) |  |
| 40–49 | 475 (19.4) | 131 (21.3) | 344 (18.8) |  |
| 50–64 | 643 (26.3) | 162 (26.4) | 481 (26.3) |  |
| 65–74 | 535 (21.9) | 122 (19.9) | 413 (22.5) |  |
| ≥75 | 101 (4.1) | 29 (4.7) | 72 (3.9) |  |
| Employment status |  |  |  | .127 |
| Employed ^b^ | 1,290 (52.7) | 346 (56.4) | 944 (51.5) |  |
| Not employed, not seeking work ^c^ | 415 (17.0) | 89 (14.5) | 326 (17.8) |  |
| Not employed, seeking work | 194 (7.9) | 50 (8.1) | 144 (7.9) |  |
| Retired | 547 (22.2) | 129 (21.0) | 418 (22.8) |  |
| Marital status ^a^ |  |  |  | .865 |
| Married or living with partner | 1,658 (67.8) | 412 (67.1) | 1,246 (68.0) |  |
| Unmarried ^d^ | 770 (31.5) | 198 (32.2) | 572 (31.2) |  |
| Decline to answer | 224 (9.2) | 4 (0.7) | 14 (0.8) |  |
| Household income ^a, e^ |  |  |  | .700 |
| Low | 518 (21.2) | 140 (22.8) | 378 (20.6) |  |
| Medium | 955 (39.0) | 237 (38.6) | 718 (39.2) |  |
| High | 749 (30.6) | 184 (30.0) | 565 (30.8) |  |
| Decline to answer | 224 (9.2) | 53 (8.6) | 171 (9.3) |  |
| Level of education |  |  |  | **.026** |
| Less than high school ^f^ | 242 (9.9) | 74 (12.1) | 168 (9.2) |  |
| High school | 1,331 (54.4) | 320 (52.1) | 1,011 (55.2) |  |
| Degree | 735 (30.0) | 181 (29.5) | 554 (30.2) |  |
| Master’s degree | 136 (5.6) | 37 (6.0) | 99 (5.4) |  |
| Declined to answer | 2 (0.1) | 2 (0.3) | 0 (0.0) |  |
| Current region of residence |  |  |  | **<.001** |
| North | 1,142 (46.7) | 248 (40.4) | 894 (48.8) |  |
| Centre | 541 (22.1) | 124 (20.2) | 417 (22.8) |  |
| South | 763 (31.2) | 242 (39.4) | 521 (28.4) |  |
| Health insurance type |  |  |  | .203 |
| Public | 1,751 (71.6) | 442 (72.0) | 1,309 (71.5) |  |
| Private | 299 (12.2) | 84 (13.7) | 215 (11.7) |  |
| Unsure | 396 (16.2) | 88 (14.3) | 308 (16.8) |  |

Chronic cough was defined as daily cough for ≥ 8 weeks. Values are presented as n (%). Statistically significant *P* values (< .05) are shown in **bold**.

^a^ Age, gender, marital status, and household income were used as criteria in propensity score matching and thus would not be expected to differ significantly by chronic cough status.

^b^ ‘Employed’ category includes individuals in full-time, part-time, or self-employment.

^c^ ‘Not Employed, not seeking work’ category includes individuals who are homemakers, students, on long-term disability, or otherwise not employed and not looking for any kind of work.

^d^ ‘Unmarried’ category includes individuals who are single, never married, divorced, or separated.

^e^ Income groups were defined according to the same income categories used in the NHWS 2020 survey for Italy. Low, <€20,000; medium, €20,000–39,999; high, ≥€40,000.

^f^ ‘Less than high school’ category includes individuals with no education, or with elementary or middle school education.
